# Predicting the likelihood and amount of fading, fixed, flourishing, and flexible positive and negative affect of autobiographical memories

**DOI:** 10.3758/s13421-023-01507-2

**Published:** 2024-01-08

**Authors:** Sophie Hoehne, Daniel Zimprich

**Affiliations:** Department of Developmental Psychology, Institute of Psychology and Education, Ulm University, Albert-Einstein-Allee, 47, 89081 Ulm, Germany

**Keywords:** Autobiographical memory, Fading affect bias, Social rehearsal, Subjective wellbeing, Emotional change

## Abstract

The emotions attributed to an event can change from occurrence to recall. Autobiographical memories (AMs) exhibit fixed affect (i.e., no change in emotionality), fading affect (i.e., a decrease in emotional intensity), flourishing affect (i.e., an increase in emotional intensity), and flexible affect (i.e., change of valence). Mixed-effects multinomial models were used to predict the likelihood of the different affect change categories. Mixed-effects regression models were used to predict the amount of emotional change within each category. Predictors at the event-level were initial intensity, social rehearsal, and recall frequency. Predictors at the participant-level were components of subjective wellbeing. Analyses were based on 1,748 AMs reported by 117 young participants in response to 16 event cues. Frequency biases, and biases in the amount of change across positive and negative emotionality, were found for all types of emotional change. Specifically, there was more fading of negative (29.98%) than positive affect (11.90%), more flourishing of positive (34.27%) than negative affect (9.61%), and more AMs changing from negative to positive valence (13.33%) than vice versa (3.95%). These biases were also evident in the amount of change within the categories. Moreover, slightly more AMs remained fixed in positive (49.89%) than negative affect (47.08%). Both event and participant level predictors were significantly associated with the likelihood of different affect change categories and the amount of emotional change within the categories. The present findings highlight the importance of considering the different ways in which AMs change emotionally from occurrence to later recall.

## Introduction

When recalling an autobiographical memory (AM), individuals tend to reinterpret the event in the light of their current situation (Peters et al., 2022). Therefore, their emotional appraisal of life events can change across time, that is, from event occurrence to the present. To date, most research on the emotional change of AMs over time has been concerned with the *fading affect bias* (FAB), which describes the phenomenon that the intensity of negative emotions attributed to negative life-events fades faster and more strongly over time than the intensity of positive emotions attributed to positive life-events (Walker et al., 1997). However, in addition to fading affect, AMs can also exhibit fixed, flourishing, and flexible affect (see below) (e.g., Gibbons & Rollins, 2016; Ritchie et al., 2009), and it has only recently been shown that these other types of AMs’ emotional change also demonstrate frequency biases (see below) (Hoehne, 2023). Furthermore, Hoehne (2023) examined the effect of multiple predictors, namely, initial intensity, frequency of social rehearsal, vividness, and individual levels of rumination and reflection, on the likelihood of the different *affect change categories*. The aim of the present study was to replicate and extend Hoehne’s (2023) findings regarding frequency biases and predictors of AMs’ emotional change. In addition, the present study examined not only the probability of an AM falling into a particular affect change category, but also the strength of the emotional change within the respective category. As participants rated their perceived emotionality at event occurrence from a retrospective perspective, *perceived emotional change* of AMs from occurrence to recall was investigated.

### Fading affect bias and other frequency biases in AMs’ emotional change

Emotion is an important (Holland & Kensinger, 2010) and time-sensitive feature of autobiographical memories “that can be re-experienced, resolved or reinterpreted depending upon the social-cognitive forces in a person’s environment” (Walker & Skowronski, 2009, p. 3). To specify, emotionality has two dimensions, valence and intensity (e.g., Talarico et al., 2004; Wolf et al., 2021). Valence indicates whether something is perceived as emotionally positive or negative (or neutral, which is treated here as a zero negative and positive affect) and is therefore a categorical variable. Intensity, on the other hand, is a continuous variable, that encompasses the strength of a negative or positive emotional experience. To illustrate, one may experience an event that one generally, but not exclusively, perceives as pleasant. The valence of emotions felt in connection with the event will therefore be predominantly positive, but some aspects of the event may evoke emotions with negative valence at the same time. In addition to being perceived as having positive and/or negative valence, the strength of these emotions is described by their emotional intensity. For instance, the event may have been perceived as moderately positive, but at the same time weakly negative. Any event or memory can evoke both positively and negatively valenced emotions of varying intensity.

Both the intensity of positive and/or negative valenced emotions associated with an event can change over time. In particular, the (positive and/or negative) emotionality of an event may be perceived as less intense when recalled. Such a decrease in emotional intensity is referred to as *fading affect* (Ritchie et al., 2009). Hoehne (2023) extended the concept by additionally distinguishing between *fading positive affect* and *fading negative affect,* depending on the valence of the emotions that have decreased in intensity. Similarly, the negative or positive emotionality of an event may be perceived as more intense when recalled, which is referred to as *flourishing positive* or *negative affect* (Hoehne, 2023; Ritchie et al., 2009). In addition, if the intensity of the positive or negative emotionality of AMs does not change from the occurrence of the event to its recall, the AM exhibits *fixed positive* or *negative affect* (Hoehne, 2023; Ritchie et al., 2009). Finally, some AMs change their primary valence over time. That is, an event that was initially perceived as predominantly pleasant may be remembered as predominantly unpleasant at recall or vice versa. This type of change is referred to as *flexible positive affect* in the case of a change from positive to negative valence, or *flexible negative affect* in the case of a change from negative to positive valence (Hoehne,^ 2023^; Walker & Skowronski, 2009).

A lot of research has been conducted regarding fading affect. Most importantly, it has been shown that across a variety of different methods for collecting AMs, as well as across different samples and cultures (for an overview, see Skowronski et al., 2014), the positive affect intensity attributed to personal events tends to fade less strongly and less quickly from event occurrence to recall than the negative affect intensity attributed to personal events. This phenomenon is referred to as the *fading affect bias* (FAB) (Walker et al., 1997). Concerns that the FAB may be a methodological artefact have been addressed by Ritchie et al. (2009), among others, who found in four separate studies that the FAB could not be explained by the AM’s emotion activation level, i.e., better recall of pleasant than unpleasant events, nor by participants’ dispositional mood or retrospective bias. Given the accumulating evidence, the FAB is considered a robust and reliable phenomenon of autobiographical memory (Muir et al., 2022). In terms of the potential functional value of a FAB, Walker and Skowronski (2009) argued that it reflects a coping mechanism that supports a positive self-concept by helping to regulate emotions and can thus be seen as an underlying mechanism of the emotion-regulating, self-serving functionality of autobiographical memory.

Most commonly, the FAB has been examined by calculating difference scores between emotionality ratings from the perspective at event occurrence and the current perspective (e.g., Muir et al., 2022; Ritchie et al., 2006, 2009) to compare the amount of change in negative emotionality with the amount of change in positive emotionality over time. Importantly, it was not just the memories that had faded that contributed to this score, but all the AMs that a participant reported. On average across all of a participant’s AMs, negative emotionality decreased in intensity over time more than positive emotionality, which is referred to as the FAB. Ritchie et al. (2009) aimed to compare not only the amount of change in positive and negative intensity, but also the number of AMs that showed fading affect. As not all memories showed fading, Ritchie et al. (2009) labelled an increase in AMs’ emotional intensity as flourishing affect and no change as fixed affect. Previously, in their study of emotional change in dreams, Ritchie and Skowronski (2008) had already found that not all dreams faded in affect, but that most dreams maintained their initial intensity, while very few also increased in emotional intensity over time. Shortly after Ritchie et al. (2009), Walker and Skowronski (2009) published an article that looked more closely at flourishing and fixed affect. They also described the case of valence switching, which they called flexible affect. Since then, two studies have examined all four categories of affect change in detail (Gibbons & Rollins, 2016; Hoehne, 2023). Otherwise, studies have mostly included the frequency of all four affect change categories in the descriptive statistics (e.g., Rollins et al., 2018) or excluded from the analysis AMs that exhibited a type of change other than fading affect (e.g., Gibbons et al., 2021). However, in order to better understand how AMs’ emotionality changes over time and how these processes might be related to emotion regulation, it is necessary to look more closely at other forms of emotional change in AMs apart from fading affect.

The few studies that have looked at all four types of emotional change in AMs have consistently found frequency biases in fixed affect, flourishing affect, and flexible affect as well (Gibbons & Rollins, 2016; Hoehne, 2023; Ritchie et al., 2009). Specifically, these studies showed that: (1) there is more fading of negative than positive affect (i.e., the FAB); (2) positive intensity stays fixed more often than negative intensity; (3) positive intensity increases (flourishes) more often than negative intensity; and (4) more of the initially negative events become positively valenced memories than vice versa. These findings are surprising given the common notion that “bad is stronger than good” (Baumeister et al., 2001), suggesting that negative information is more powerful than positive information (e.g., bad feedback). However, autobiographical memory tends to be biased in a positive direction and is therefore considered to be part of an emotion-regulating self-system that works to promote a positive sense of self and to maintain an optimistic view of the world and the future (Walker & Skowronski, 2009). Not only the FAB, but also the frequency biases in fixed, flourishing, and flexible affect, can be seen as mechanisms underlying this emotion-regulating, self-serving functionality of autobiographical memory.

Following this line of reasoning, the four frequency biases, but especially the FAB, have been linked to contemporary theories of emotion and motivation. For example, the mobilization-minimization hypothesis (Taylor, 1991) proposes that emotional events should initially be followed by strong reactions, that is, a mobilization of an individual’s physiological, cognitive, and social systems to cope with the immediate consequences of the event. After some time, however, the individual should begin to minimize the long-term consequences of the event by activating a series of counter-processes (e.g., causal reasoning). The theory suggests that both mobilization and minimization processes should be stronger for negative than for positive emotionality (Skowronski et al., 2014). The theory, in particular, supports the FAB and the bias in flexible affect, as more fading and flexible affect in negative compared to positive emotionality indicate stronger minimization processes in the negative emotionality of an event. However, the biases in flourishing and fixed affect are also supported by this theory, as they indicate that positive emotionality is more likely not to be minimized.

From a different, but related perspective, the broaden-and-build theory of positive emotions (Fredrickson, 1998) suggests that positive emotions lead to altered patterns of action and decision-making, such as a broadened scope of attention, increased cognitive flexibility, and creativity. These altered patterns of action and decision-making, in turn, are thought to build long-term psychological, physical, and social resources (Conway et al., 2013). In addition, the broaden-and-build theory proposes that negative emotions, such as fear or anxiety, are primarily associated with narrowed and focused attention, whereas positive emotions help to broaden attention and thus often have a countervailing effect on negative emotions (Fredrickson, 1998). This suggestion that positive emotions help to build themselves up over time, but counteract negative emotions, fits with the assumption of a fading, flourishing, fixed, and flexible affect bias, as these biases imply that positive emotionality increases or remains fixed more often, while negative emotionality decreases.

Finally, the self-enhancement theory (Sedikides & Alicke, 2019) proposes that motives of self-enhancement and self-protection, manifested in five so-called key-pillars, motivate human behavior and cognition. One of these key-pillars is selective self-memory, which proposes that individuals should have good memories of their virtues but poor memory of their faults. Again, all four biases fit into this theory, because as a result of these biases, our autobiographical memory, and therefore our sense-of-self is pulled away from the negative but pushed towards the positive (see Hoehne, 2023).

In summary, all three theories mentioned above would suggest that in AMs, negative emotionality should decrease more often than positive emotionality over time, while positive emotionality should not change or increase more often than negative emotionality, which is consistent with the observation of the different affect change biases. Given the theories and previous evidence, the first hypothesis of the present study was that biases would be found for all four affect change categories. Specifically, it was hypothesized that there would be more fading and flexible affect for negative emotionality, but more fixed affect and flourishing affect for positive emotionality (H1).

One might wonder how a change in emotionality could be related to the phenomenological quality or reliability of an AM. Re-evaluating an event in a different light does not necessarily mean that one is remembering the event differently, but could also be the result of experiencing its consequences, for instance (Gibbons & Rollins, 2016), or receiving positive feedback from a listener (Hoehne, 2023; Skowronski et al., 2004). However, previous studies have found that a decrease, but also an increase, in the emotional intensity of an AM is associated with a loss of AM vividness compared to no change (Hoehne, 2023; Lindeman et al., 2017; Ritchie & Batteson, 2013). In general, it remains relatively unexplored how different affect change categories relate to different characteristics of the memory or the individual remembering the event.

### Predicting AMs’ emotional change

More recently, Hoehne (2023) has investigated the influence of several predictors on the different types of perceived emotional change in AMs. Specifically, it was examined how the memory characteristics of initial intensity, social rehearsal, and vividness influence an AM’s probability to show a specific type of emotional change, and how the individual characteristics rumination and reflection influence individuals’ probability to report AMs from the different affect change categories. Participants were asked to recall up to 12 memories in response to different emotion-words and to rate separately the positive and negative intensity of each AM at the time of event occurrence as well as from a current perspective. Based on these ratings, each AM was assigned to one of four affect change categories regarding positive and negative emotionality ratings. Initial intensity, social rehearsal, rumination, and reflection yielded partly unexpected results (see below). The present study aimed to explore these variables in more detail by replicating and extending Hoehne’s (2023) findings.

Initial intensity is the only predictor variable that, apart from Hoehne (2023), has previously been examined in the context of all four affect change categories. Gibbons and Rollins (2016) asked participants to provide brief descriptions of unpleasant and pleasant memories, and to rate the emotionality (negative to positive) of each AM at the time of event occurrence and from a current perspective. Based on the difference scores between these two emotionality ratings, they assigned each AM to one of the four affect change categories and analyzed them separately for initially positive and initially negative valence. The lowest initial intensity ratings were found for flourishing affect memories, followed by flexible, fading, and fixed affect memories. Hoehne’s (2023) findings are broadly consistent, but it was found that regardless of valence, flexible affect memories were associated with relatively high initial intensity ratings, higher than those of fixed affect memories, whereas Gibbons and Rollins (2016) suggested that flexible affect memories may be associated with low initial intensity ratings. This may be due to the operationalization of flexible affect in Hoehne (2023), where participants had to actively choose whether and in which direction their memory changed valence, rather than being defined as a difference score as in Gibbons and Rollins (2016), where flexible affect becomes more likely the closer the initial event intensity is to neutral. The present study used the same procedure as Hoehne (2023), with a different sample and a different technique for collecting memories, in an attempt to replicate these findings. It was hypothesized that the effects of initial intensity would show the same pattern as in Hoehne (2023), that is, regardless of valence, higher levels of initial intensity would be associated with less flourishing affect but more fading and flexible affect in an AM compared to no change (H2).

Social rehearsal, that is, sharing an AM with others, has been extensively investigated in the context of the FAB. Skowronski et al. (2014), who found evidence for a moderating role of social rehearsal on the FAB (Skowronski et al., 2004), argued that social sharing promotes positivity through cognitive resources that work to structure the memory (e.g., reflecting on it), as well as through elicitation and supportive feedback from the listener (e.g., Muir et al., 2015, 2019), which may help to promote the positive emotionality of positive memories. More specifically, Skowronski et al. (2004) found that communicating an AM to others led to a reduced decrease or even an increase in the emotional intensity of positive AMs and a stronger affective fading of negative AMs. Hoehne (2023) found evidence that high levels of social rehearsal led to a decreased likelihood of an AM exhibiting fading and an increased likelihood of exhibiting flourishing affect in relation to the positive emotionality of AMs, which is consistent with Skowronski et al.’s (2004) findings. However, Hoehne (2023) did not find evidence for a stronger fading of negative emotionality in AMs that were frequently shared. On the contrary, Hoehne (2023) reported that social rehearsal led to a lower probability for an AM to show fading of negative emotionality. What may explain these different findings is that, unlike Skowronski et al. (2004), Hoehne (2023) used separate scales to measure each AM’s positive and negative emotionality. To clarify, the decrease in the negative emotional intensity of the AM after social disclosure in Skowronski et al.’s (2004) study could be due to three different processes, which cannot be distinguished from each other due to the use of only one scale to measure the emotionality of the AM. First, the AM could have been rated as less negative because its negative aspects were perceived as less negative, or second, its positive aspects were perceived as more positive, or third, both processes come into play. Consistent with Muir et al.’s (2019) finding that positive facilitation (rather than negative inhibition) is a relevant aspect of listeners’ responses to enhance FAB following social disclosure, Hoehne (2023) suggested that the benefits of social rehearsal operate primarily through the enhancement of positive emotionality rather than, as some authors have suggested (e.g., Cohen & Wills, 1985), through the reduction of negative emotionality. To support this suggestion, the present study again included social rehearsal and separate scales for positive and negative emotionality in an attempt to replicate these findings. It was hypothesized that higher levels of social rehearsal would be primarily associated with an increase in positive emotionality in an AM, but not with a decrease in negative emotionality (H3).

Rumination involves unwanted and repetitive thinking about negative past events, which compels individuals to re-experience the negative emotions of the event, which, as a consequence, may maintain their strength (Ritchie et al., 2009), whereas reflection involves thinking about past events “out of philosophical love of self-exploration” (Harrington & Loffredo, 2010, p.45). Hoehne (2023) used the Rumination-Reflection Questionnaire (Trapnell & Campbell, 1999) to investigate rumination and reflection as predictors at the participant level, that is, whether individual levels of rumination or reflection influenced the likelihood of individuals reporting AMs from different affect change categories. Neither rumination nor reflection yielded significant effects. The reader may note that while the Rumination-Reflection Questionnaire (Trapnell & Campbell, 1999) captures the general tendency to ruminate (or reflect) on past events, it does not specifically capture the individual’s tendency to ruminate on AMs. When rumination is investigated as a memory characteristic as in Marsh et al. (2019), it was associated with reduced fading of the negative emotionality of AMs. In line with the common notion that thinking about life events changes the way they are evaluated, it stands to reason that rumination and reflection should be associated with perceived emotional changes in AMs, but perhaps as a memory characteristic rather than an individual trait. One variable that might combine rumination and reflection, however, without specifying the type of thinking, is the frequency with which an event is recalled, also referred to as (overall) private rehearsal. Recently, Muir et al. (2022) found that overall private rehearsal of negative AMs was associated with less fading of the negative emotionality of that AM. This finding adds to the body of evidence from studies that have found an association between increased frequency of private rehearsal and a reduced FAB (Ritchie et al., 2006, 2015; Walker et al., 2009). The present study aimed to build on this evidence and extend Hoehne’s (2023) findings by investigating how the frequency of event recall – both social (see above) and private – as a characteristic of an AM is related to that AM’s probability exhibiting a particular type of emotional change. It was hypothesized that higher levels of recall frequency would be associated with less fading negative affect in an AM, but would also be associated with other types of emotional change in AMs (H4).

Several individual characteristics examined in previous studies have been found to reduce the FAB and its positive effects, such as depression (Walker et al., 2003), anxiety (Walker et al., 2014), and narcissism (Ritchie et al., 2015). More recently, the FAB has also been investigated from a positive psychology perspective, with an increased FAB found in those with higher individual levels of grit (Walker et al., 2020). One particular individual characteristic that may be meaningful in relation to individuals’ perceived emotional changes of AMs is subjective wellbeing (SWB). SWB has been operationalized by Diener (1984) as high levels of positive affect combined with low levels of negative affect and high levels of satisfaction with life. As such, measures of SWB combine adaptive personality traits, that is, positive affect and life satisfaction, with maladaptive personality traits, that is, negative affect, making the construct promising for research on AMs’ emotional change, which has so far mostly focused on maladaptive individual difference measures. In order to extend Hoehne’s (2023) findings and to further investigate individual characteristics in relation to the likelihood of individuals reporting AMs from the different affect change categories, the present study included the predictor variables life satisfaction, positive affect and negative affect as characteristics of the individual. It was hypothesized that higher levels of individual wellbeing would be associated with more positive and less negative changes in a participant’s AMs (H5).

One limitation of Hoehne’s (2023) study (and previous studies of the four affect change categories) is that it used a categorical statistical approach, which allowed the *probabilities* of the different affect change categories to be modelled. However, a categorical approach does not allow for the investigation of how predictors affect the *strength* or *amount* of emotional change of an AM. Typically in FAB research, however, studies investigate how specific predictors relate to the strength of affective fading and whether these effects differ between positive and negative valence (e.g., Marsh et al., 2019; Ritchie et al., 2009; Walker et al., 2014). In order to provide a more complete picture, the present study investigated not only how specific predictors relate to the probability of the different affect change categories, but also how the same predictors relate to the amount of change within each category. In addition, the present study extended the first hypothesis in that it was hypothesized that biases in fading, flourishing, flexible, and fixed affect would be evident not only in the frequency of the different categories across positive and negative emotionality, but also in the amount of change within the categories. Specifically, it was hypothesized that not only would there be more, but also stronger fading and flexible affect for negative emotionality, and stronger flourishing affect for positive emotionality (extended H1).

### The present study

The emotions attributed to an event can change depending on the situation at the time of recall (Peters et al., 2022; Walker & Skowronski, 2009). In this sense, AMs may exhibit fading, fixed, flourishing, and flexible affect, each of which shows a bias in their frequency across positive and negative emotionality of AMs (Gibbons & Rollins, 2016; Hoehne, 2023; Ritchie et al., 2009). The aim of the present study was to replicate and extend Hoehne’s (2023) findings regarding frequency biases and predictors of the four affect change categories, in order to gain more knowledge about the less prominent types of AMs’ emotional change other than fading affect.

Gibbons and Rollins (2016) argued that one reason why affect change categories other than fading affect have often been neglected to date may be the low frequency with which they have emerged in previous studies and the recall procedures that may have favored the retrieval of highly intense memories. Hoehne (2023) therefore used a recall technique that specifically elicited a range of memories of different valence and intensity, and found relatively high rates of affect change categories other than fading affect. In the present study, yet another type of memory elicitation was used to sample AMs of different valence and intensity. Participants were given positive and negative events and were asked to indicate whether they had experienced the event in question. In the next step, participants were asked to rate the emotionality of the events they had experienced. In acknowledging that positive and negative affect are distinct dimensions of human emotional experience (Diener & Emmons, 1985), participants were asked to rate the positive and negative emotional intensity of each AM separately (for a similar procedure see Ritchie et al., 2006). Analyzing the emotional change of positive and negative intensity separately allowed the influence of each predictor on the change of positive and negative emotionality of AMs to be examined separately.

Only young participants were included in the present study. The reason for this is that, due to a phenomenon known as the *age-related positivity effect* (Comblain et al., 2005; Gallo et al., 2011; Kennedy et al., 2004), younger and older participants are likely to differ in their emotional ratings of AMs.

Everyday remembering includes memories that vary in several characteristics, such as their initial intensity or their frequency of social rehearsal and recall, which are represented at the event level. Furthermore, AMs and their affective change differ between participants due to specific characteristics of the individual, such as their level of wellbeing, which are represented at the participant level (e.g., Walker et al., 2003). When participants recall a larger number of events, a multilevel analysis is appropriate to account for the fact that memories are nested within participants, i.e., the AMs of one participant may have something in common because they were reported by the same individual. In addition to accounting for the hierarchical structure of the data, multilevel models provide the opportunity to examine predictors at two different levels: the level of events (or AMs) and the level of participants. Specifically, this means that in the present study we were able to test whether the emotional change of an AM could be predicted by characteristics of the memory (initial intensity, social rehearsal, frequency of recall) and characteristics of the remembering individual (wellbeing). A reference category had to be chosen for the different models to be estimated in the present study. Similar to Hoehne (2023), fixed affect was chosen as the reference category. In doing so, the present study investigated whether between-person differences in individual characteristics, as well as within-person differences in memory-characteristics, could explain why certain AMs (a) fall into a particular category of emotional change, and (b) vary in the amount of emotional change within the respective category, as compared to remaining fixed in affect.

## Method

### Participants

The final sample was comprised of 117 young adults^1^. Three participants had to be excluded prior to the analyses, because they declined to give informed consent. On average, participants were 22 years old (*SD* = 2.16 years), ranging from 18 to 29 years of age. The sample consisted of 82.9% female participants, while 1.7% of them reported being gender diverse. Participants were recruited through e-mail, promotional flyers, and word of mouth.

### Procedure

Data were collected online (http://www.unipark.de). The study was conducted in accordance with the ethical principles of the Declaration of Helsinki and the collection and further processing of the research data took place in anonymized form. After reading through the study information and giving their informed consent, participants provided demographic (e.g., age, sex, education, marital status) and subjective health information. Subsequently, they were administered the Satisfaction with Life Scale (SWLS; Diener et al., 1985) and the Positive and Negative Affect Schedule (PANAS; Watson et al., 1988). Next, AMs were cued with 16 event cues (see below).

#### Memory cues

Autobiographical memories were elicited using cues for distinct life events from the Autobiographical Memory Questionnaire (AMQ; Denkova et al., 2012). The AMQ is a list of 115 verbal cues to life events that vary in valence and intensity. For the present study, eight positive (being proud of oneself; surprising oneself positively; overcoming a fear; trying a new hobby; falling in love; hosting a party; meeting a significant other for the first time; attending a friends birthday party) and eight negative event cues (getting rejected; feeling very ashamed; being mocked by others; argument with parents; being lied to; failing at school/work; fighting with your best friend; embarrassing yourself) were chosen to vary in emotional intensity, to be likely to have been experienced by most participants, and to be potentially self-relevant^2^. The valence manipulation was successful, as AMs reported in response to positive event cues had, on average, higher positive and lower negative intensity ratings (positive then: *M* = 5.33, *SD* = 3.20; positive today: *M* = 5.46, *SD* = 3.13; negative then: *M* = 1.85, *SD* = 3.03; negative today: *M* = 1.64, *SD* = 2.90) than AMs reported in response to negative event cues (positive then: *M* = 1.32, *SD* = 2.84; positive today: *M* = 2.06, *SD* = 3.09; negative then: *M* = 5.92, *SD* = 3.14; negative today: *M* = 4.63, *SD* = 3.28).

Event cues were presented in a randomized order. After the presentation of each cue-word, participants were asked if they could think of an event from their past that matched the event cue. If they remembered a matching event, they were asked to provide a brief description of the event. Participants were instructed that events should be specific and older than one year. After all events had been described, participants were presented with each of their event descriptions again in a randomized order and answered several questions about each event. Questions included age at the time of the event, frequency of social rehearsal, frequency of recall, positive and negative emotionality then and now, and whether the event had changed in valence from then (occurrence) to now (test).

### Measures

#### Memory characteristics

Participants rated the *emotionality* of each event from the perspective of the event occurring and from the perspective of the event being remembered, that is now. The ratings were made separately for positive and negative emotionality. The four resulting items were presented in a randomized order for each memory. Responses were made on a scale ranging from *not at all* (1) to *very much* (7). Ratings of emotionality at the time of event occurrence were used to measure *initial intensity*. When examining change in positive emotionality, positive intensity at event occurrence was used as a measure of initial intensity, whereas when examining change in negative emotionality, negative intensity at event occurrence was used as a measure for initial intensity. In addition, participants rated the frequency of *social rehearsal* for each event on one item (“How often do you talk to someone about the event?”). Responses were made on a 7-point Likert-scale ranging from *almost never* (1) to *very often* (7). Participants also rated the *frequency of recall* for each event in an item (“How often do you recall the event in your everyday life?”). Answers were given on a 7-point Likert-scale ranging from *almost never* (1) to *very often* (7). Finally, in order to distinguish *flexible affect* memories from other memories, an additional question (Q1) was formulated to capture the change in valence (“Would you say that your feelings related to this event have changed?”). Three possible answers were given: *Yes, they were positive before but negative now*; *Yes, they were negative before but positive now*; *No*.

#### Satisfaction with Life Scale (SWLS)

The SWLS (Diener et al., 1985) is a five-item questionnaire measuring life satisfaction, which is considered the cognitive-evaluative component of subjective wellbeing (Diener, 1984). For the present study, the German translation by Janke and Glöckner-Rist (2014) was used. Participants rated how much they agreed with five different statements regarding domain-specific and global life satisfaction on a 7-point Likert-type scale ranging from *strongly disagree* (1) to *strongly agree* (7). An example item is: “In most ways me life is close to my ideal.” In the present sample, Cronbach’s alpha for the SWLS was .86. Participants were moderately satisfied with their life on average (*M* = 4.82, *SD* = 1.26).

#### Positive and Negative Affect Schedule (PANAS)

The PANAS (Watson et al., 1988) is a widely used instrument for measuring emotional states. It has often been used to capture the affective component of subjective wellbeing (Diener, 1984). For the present study, the German translation by Breyer and Bluemke (2016) was used. The questionnaire consists of 20 adjectives, each describing different emotions and feelings, for which participants were asked to indicate the intensity of each one on a 5-point Likert-type scale ranging from *very slightly/ not at all* (1) to *extremely* (5). The positive and negative affect subscales each consist of ten adjectives. Example of positive affect items are “enthusiastic” or “inspired” and for negative affect items “upset” or “nervous”. Participants were asked to rate the intensity of the different emotions and feelings based on *how they feel in general*. In the present sample, the Cronbach’s alpha for the positive affect subscale was .86, and the Cronbach’s alpha for the negative affect subscale was .88. On average, participants rated their positive affect as moderately intense (*M* = 3.19, *SD* = 0.66) and their negative affect as weakly intense (*M* = 1.96, *SD* = 0.72).

### Statistical approach

With the aim of examining both qualitative and quantitative changes in positive in negative affect of AMs, we conducted two different types of analyses. For both analyses, each AM had to be classified into one of four affect change categories in terms of positive and negative emotionality ratings. Therefore, difference scores between emotionality ratings at event occurrence and event recall were constructed for each AM separately for positive and negative emotionality ratings. If positive intensity decreased, an AM was categorized as *fading positive affect*. Similarly, if positive intensity increased, an AM was categorized as *flourishing positive affect,* and as *fixed positive affect* when positive intensity did not change. The same was done for negative emotionality ratings. Finally, if the AM was rated as having changed valence (Q1), it was categorized as *flexible positive affect* if it changed from positive to negative, or *flexible negative affect* if it changed from negative to positive.

More specifically, Let ${d}_{ijk}$ denote the difference in emotional intensity of memory $j$ ($j$ = 1… *n*_*i*_) in person $i$ ($i$= 1…* N*) regarding valence $k$ ($k$ = N [*negative*], P [*positive*]) between event occurrence and at current (i.e., ${d}_{ijP}= {y}_{ijP}-{x}_{ijP}$, where ${y}_{ijP}$ is the positive emotional intensity rating at current and ${x}_{ijP}$ is the positive emotional intensity rating at event occurrence). Based on ${d}_{ijP}$, three categories of changes can be distinguished:

1 $${u}_{ijP}=\left\{\begin{array}{lc}0\;\;\;\;\;\mathrm{if}\;\;\;{d}_{ijP}=0\;\;\;\;\;\;:\;\;\;\;\;\;\;\;\;\; \mathrm{fixed\; affect} \, \\ 1\;\;\;\;\; \mathrm{if}\;\;\;{d}_{ijP}>0\;\;\;\;\;\;:\;\;\;\;\;\;\;\;\;\;\mathrm{flourishing\; affect}\\ -1\;\; \mathrm{if}\;\;\;{d}_{ijP}<0\;\;\;\;\;\;:\;\;\;\;\;\;\;\;\;\;\mathrm{ fading\; affect } \, \end{array}\right.$$

where $${u}_{ijP}$$ is a categorical variable that denotes whether a memory $$j$$ in person $$i$$ showed fading, fixed, flourishing or flexible positive affect. Moreover, by introducing an additional variable $${k}_{i{j}_{t}}$$ that denotes the dominating valence of memory $$j$$ in person $$i$$ at occurrence ($$t=1)$$ or recall ($$t=2)$$, flexible positive affect can be defined as

2 $${u}_{ijP}=2\; \mathrm{if }\;{k}_{i{j}_{1}}=P\; \& \;{k}_{i{j}_{2}}=N: \mathrm{flexible\; affect}$$

#### Qualitative change

For the first part of the analysis, the dependent variable consisted of the four affect change categories. All analyses were conducted separately for change of positive and change of negative emotionality. To analyze the probability of the four affect change categories, a mixed-effects multinomial model was used (Hedeker, 2003; Hedeker & Gibbons, 2006), which allows for modelling the probability of the unordered categories^3^ of a dependent variable. A reference category needs to be selected to estimate a multinomial regression model. Similar to Hoehne (2023), fixed affect was chosen as the reference category. Consequently, the multinomial regression can be thought of as the simultaneous estimation of logistic regression models for the comparisons between (1) fading affect versus the reference category of fixed affect, (2) flourishing affect versus fixed affect, and (3) flexible affect versus fixed affect – but with increased statistical power compared to conducting three logistic regression analyses (Stroup, 2012). In addition, to account for the two-level structure of the data in the present study (event level, participant level), the multinomial regression model was extended to include random intercept effects (see Hedeker, 2003; Hedeker & Gibbons, 2006). These random intercepts allow for the fact that, typically, individuals differ in the probabilities with which they report AMs with flourishing, fading, and flexible affect change, which (taken together) also implies that they differ in the probability of reporting fixed affect. A much more detailed and accessible description of the mixed-effects multinomial regression model is provided by Zimprich and Wolf (2018).

#### Quantitative change

For the second part of the analysis, to analyze the amount of affective change in the different categories, we used multilevel multiple regression. The reader may note that, by definition, the amount of quantitative change in fixed AMs is zero. Comparably to the multinomial model, fixed AMs served as the reference category. A dummy-coded indicator variable was created for each type of change. For example, the indicator variable for fading positive affect was coded as 1 if the AM showed fading positive affect and as 0 if it did not. Indicator variables were then included as predictors, together with their interactions with the other predictor variables^4^.

Six variables entered both the qualitative and the quantitative models as predictors of emotional change in AMs. Three of these were entered as event level variables, namely initial intensity, social rehearsal, and frequency of recall, and another three were entered as participant level variables, namely positive affect, negative affect, and life satisfaction. The event level predictors were centered within participants (group-mean centering), whereas the participant level predictors were centered across participants (grand-mean centering). This centering method allowed the event level predictors to account for only the event level variance in the outcome variables, and the participant level predictors to account for only the participant level variance in the outcome variable.

In addition, control variables were added, namely the sex of the participants, as well as the time since the event and the valence of the cue for each AM. Sex was dummy-coded as *female* (0) and *male* (1) and then grand-mean centered. Time since the event was calculated as each participant’s age at event recall minus age at event occurrence and entered into the models group-mean centered. Finally, the valence of the cue for each AM was dummy-coded as *positive* (0) and *negative* (1) and then group-mean centered before entering the model.

Data were analyzed using SAS NLMIXED (SAS Institute Inc., 2014) using Gauss-Hermite quadrature with 10 quadrature points and SAS MIXED. The SAS syntax and dataset (Hoehne & Zimprich, 2024) used in the present study are publicly available. Akaike’s Information Criterion (AIC; Akaike, 1974) was used to assess model fit. The AIC is based on minus two times the log-likelihood (-2LL) of the data in the model plus a penalty factor for additional parameters, thus rewarding parsimony. A more detailed discussion of the properties of the AIC can be found in Vrieze (2012). The alpha-level for testing statistical significance was set at .05. Additionally, R2 values were calculated on Level 1 and Level 2 for the quantitative change models using the approach proposed by Rights and Sterba (2019). For the R^2^ values in the qualitative change models, the approach of Jaeger et al. (2017) was utilized.

## Results

### Descriptive statistics

Participants retrieved 1,748 AMs in total that fit criteria for data analysis. The reader may note that this number does not equal 117 (sample size) × 16 (number of cue-words) = 1,872, which is due to a few missing values that resulted when participants were unable to think of a memory in response to some cue-words (*n* = 48) or when they did not provide emotionality ratings (*n* = 13). Additionally, two separate reviewers rated each event description as either valid or not valid (*n* = 63). Inter-rater reliability (Cohen’s kappa) was .85. On average, participants retrieved 14.94 AMs with nine memories being the lowest number of AMs reported by an individual. Participants reported slightly more AMs in response to positive (*M* = 7.69) than negative cues (*M* = 7.25) on average, but the size of this difference is negligible for the analysis to be presented below.

Reported memories were, on average, infrequently rehearsed socially (*M* = 2*.*26*, SD* = 3*.*06) and also infrequently recalled in everyday life (*M* = 2*.*68*, SD* = 3*.*12). From the perspective of event occurrence, positive emotionality was rated as significantly less intense than negative emotionality (positive then: *M* = 3*.*33*, SD* = 3.63; negative then: *M* = 3*.*78*, SD* = 3*.*69) (*t*(116) = -3.78*, p* < .001). However, from the perspective of event recall, positive emotionality was rated as significantly more intense than negative emotionality (positive now: *M* = 3*.*76*, SD* = 3.42; negative now: *M* = 3*.*04*, SD* = 3.53) (*t*(116) = 6*.*51*, p < .*001). Descriptive statistics for the four affect change categories are shown in Table 1. Overall, and as expected, there was more fading negative than fading positive affect, slightly more fixed positive than fixed negative affect, more flourishing positive than flourishing negative affect, and finally, there were more AMs that changed from negative to positive than vice versa (flexible affect). Furthermore, different affect change biases were also evident in the amount of change within each category. Within the category of fading affect, fading was stronger for negative than for positive emotionality. Similarly, flourishing was stronger for positive than for negative emotionality, and flexible affect AMs showed a stronger decrease in negative than in positive emotional intensity.

**Table 1:** Descriptive statistics for the four affect change categories of autobiographical memory reported by 117 participants in response to 16 event cues separately for positive and negative emotionality ratings

	Category		Total	Fading affect	Fixed affect	Flourishing affect	Flexible affect
Event level	Positive	N	1,748	208	872	599	69
		%	100	11.90	49.89	34.27	3.95
	Negative	N	1,748	524	823	168	233
		%	100	29.98	47.08	9.61	13.33
Participant level	Positive	Mean number	14.94	1.78	7.45	5.12	0.59
		SD	1.35	1.63	2.74	2.87	0.87
		Range	9–16	0–7	0–15	0–13	0–4
	Negative	Mean number	14.94	4.48	7.03	1.44	1.99
		SD	1.35	2.49	2.52	1.69	1.54
		Range	9–16	0–12	1–15	0–11	0–7
Mean change of emotional intensity*	Positive	Mean change	0.43	−1.41	0.00	1.95	−1.85
		SD	1.45	0.61	0.00	1.12	1.83
	Negative	Mean change	−0.74	−1.78	0.00	1.63	−2.72
		SD	1.51	0.91	0.00	0.88	1.52

^*^Emotional intensity measured on a scale from *not at all* (1) to *very much* (7)

### Qualitative change (multilevel multinomial model)

In the first part of the analysis, probabilities of the different affect change categories were examined. To start with, a baseline model (Model 0) was estimated that included only fixed and random intercepts. Parameter estimates for the comparisons of fixed affect (reference category) with fading, flourishing and flexible affect AMs for both the positive and negative affect models are shown in Table 2.

**Table 2:** Model 0 for qualitative change of positive and negative intensity; Event level: n = 1748; Participant level: N = 117

	Positive Affect Model			Negative Affect Model		
	Fading Positive Affect	Flourishing Positive Affect	Flexible Positive Affect	Fading Negative Affect	Flourishing Negative Affect	Flexible Negative Affect
*Fixed Effects*						
Intercept	−1.566*	−0.443*	−2.761*	−0.496*	−1.899*	−1.315*
*Random Effects*						
Intercept Variance	0.506*	0.531*	0.448	0.340*	1.013*	0.188
*Intercept Correlations:*						
Fading & Flourishing	0.327			0.402*		
Fading & Flexible	0.001			0.209		
Flourishing & Flexible	−0.175			−0.017		
*Model Fit*						
-2LL	3744			4154		
AIC	3762			4172		

* indicates *p* < .05 (two-tailed); † indicates *p* < .10 (two-tailed)

Reference Category: Fixed Affect

Intraclass Correlations Positive Affect Model: .19 (Fading), .20 (Flourishing), .18 (Flexible)

Intraclass Correlations Negative Affect Model: .14 (Fading), .32 (Flourishing), .08 (Flexible)

The intercept estimate for the category of fading positive affect was *β*_0*FadeP*_ = −1*.*566 (left half of Table 2). Transformed back to the probability scale^5^, the probability of fading positive affect was 0*.*109, implying that when accounting for random effects (i.e., individual differences), 10.9% of the reported memories are predicted to show fading positive affect. Transforming the intercepts for the other affect change categories from the positive affect model produces 0.335 (flourishing), 0.033 (flexible), and 0.523 (fixed). The reader may note that these probabilities differ slightly from the observed relative frequencies (Table 1), which is due to differences between marginal and conditional effects^6^. Participants reliably differed in their proportions of fading and flourishing positive, but not flexible positive affect, as indicated by the statistical (non-)significance of the random intercept variances (see Table 2). Intraclass correlations ranged from .19 (flexible positive affect) to .20 (flourishing positive affect) for the positive affect model, indicating that between 19% and 20% of the total variance in the affective change categories reflected differences between participants. There were no significant correlations between random effects.

Transforming the intercepts from the *negative* affect model (right half of Table 2) back to the probability scale produces 0*.*301 (fading), 0.074 (flourish), 0.132 (flexible), and 0.493 (fixed). Participants differed in the probabilities of fading and flourishing but not flexible negative affect AMs. Intraclass correlations ranged from .08 (flexible negative affect) to .32 (flourishing negative affect). The random effects of fading and flourishing negative affect were positively correlated, indicating that participants who reported relatively more fading negative AMs also tended to report more flourishing negative AMs.

In Model 1, control and predictor variables were added, resulting in an increase in model fit for both the positive and negative affect model (see -2LL and AIC in Table 3). As indicated by R^2^, the control and predictor variables together accounted for between 59.9% (fading positive affect) and 31.3% (flexible positive affect) of the total variance in the positive affect models and between 76.7% (fading negative affect) and 19.9% (flourishing negative affect) of the total variance in the negative affect models. In the following, the results are presented separately for each predictor variable (Table 3).

**Table 3:** Model 1 for qualitative change of positive and negative intensity; Event level: n = 1748; Participant level: N = 117

	Positive Affect Model			Negative Affect Model		
	Fading Positive Affect	Flourishing Positive Affect	Flexible Positive Affect	Fading Negative Affect	Flourishing Negative Affect	Flexible Negative Affect
*Fixed Effects*						
Intercept	−2.490*	−0.600*	−3.092*	−0.694*	−1.952*	−1.545*
Sex^a^	−0.196	−0.001	0.033	−0.142	−0.326	−0.395
Time since the Event^b^	0.059*	0.080*	−0.022	0.016	0.037	0.013
Cue Valence^b^	−0.364	−1.266*	0.373	−0.378	0.927*	−0.539†
*Within-Person Vars*						
Initial Intensity^b^	0.572*	−0.429*	0.402*	0.647*	−0.145*	0.659*
Social Rehearsal^b^	−0.222*	0.307*	−0.185	−0.022	−0.083	0.173†
Frequency of Recall^b^	−0.146	0.095†	−0.106	−0.240*	0.236*	−0.367*
*Between-Person Vars*						
Satisfaction with Life^a^	−0.060	0.230*	−0.126	0.214†	−0.220†	0.292*
Positive Affect^a^	−0.252	−0.042	0.396	−0.364†	−0.081	−0.083
Negative Affect^a^	−0.005	−0.229	0.489*	−0.066	0.153	−0.403*
*Random Effects*						
Intercept Variance	0.984*	0.589*	0.431	0.891*	0.877*	0.578*
*Intercept Correlations:*						
Fading & Flourishing		0.189			0.375*	
Fading & Flexible		0.401			0.758*	
Flourishing & Flexible		−0.103			0.242	
*Model Fit*						
-2LL		3256			3477	
AIC		3328			3549	
R²	0.599	0.539	0.313	0.767	0.199	0.677

^*^ Indicates *p* < .05 (two-tailed); † indicates *p* < .10 (two-tailed)

^a^ Variable is centered between persons

^b ^Variable is centered within persons

R^2^calculated according to Jaeger et al. (2017)

Reference category: Fixed affect

#### Event level predictors

##### Initial intensity

In both the positive and the negative affect model, higher initial intensity ratings were associated with an increased probability of fading (OR(positive) (for standardized factor change): 3.86; OR(negative): 4.82)^7^ and flexible affect (OR(positive): 2.58; OR(negative): 4.96) but a decreased probability of flourishing affect (OR(positive): 0.36; OR(negative): 0.70) compared to staying fixed in affect.

##### Social rehearsal

In the positive affect model, higher levels of social rehearsal were associated with a decreased probability of fading affect (OR: 0.75), but an increased probability of flourishing affect (OR: 1.50) compared to staying fixed in affect. In the negative affect model, social rehearsal did not show significant effects.

##### Frequency of recall

In the positive affect model, frequency of recall did not show significant effects. In the negative affect model, higher levels of recall frequency were associated with a decreased probability of fading (OR: 0.72) and flexible affect (OR: 0.61), but an increased probability of flourishing affect (OR: 1.38) compared to staying fixed in affect.

#### Participant level predictors

##### Satisfaction with life

In the positive affect model, individuals with higher life satisfaction had an increased probability of reporting flourishing compared to fixed affect AMs (OR: 1.33). In the negative affect model, individuals with higher life satisfaction had an increased probability of reporting flexible compared to fixed affect AMs (OR: 1.43).

##### Positive affect

No significant effects of positive affect were found.

##### Negative affect

In the positive affect model, individuals with higher levels of negative affect had an increased probability of reporting flexible compared to fixed affect AMs (OR: 1.42). In the negative affect model, individuals with higher levels of negative affect had a decreased probability of reporting flexible compared to fixed affect AMs (OR: 0.75).

**Fig. 1:**
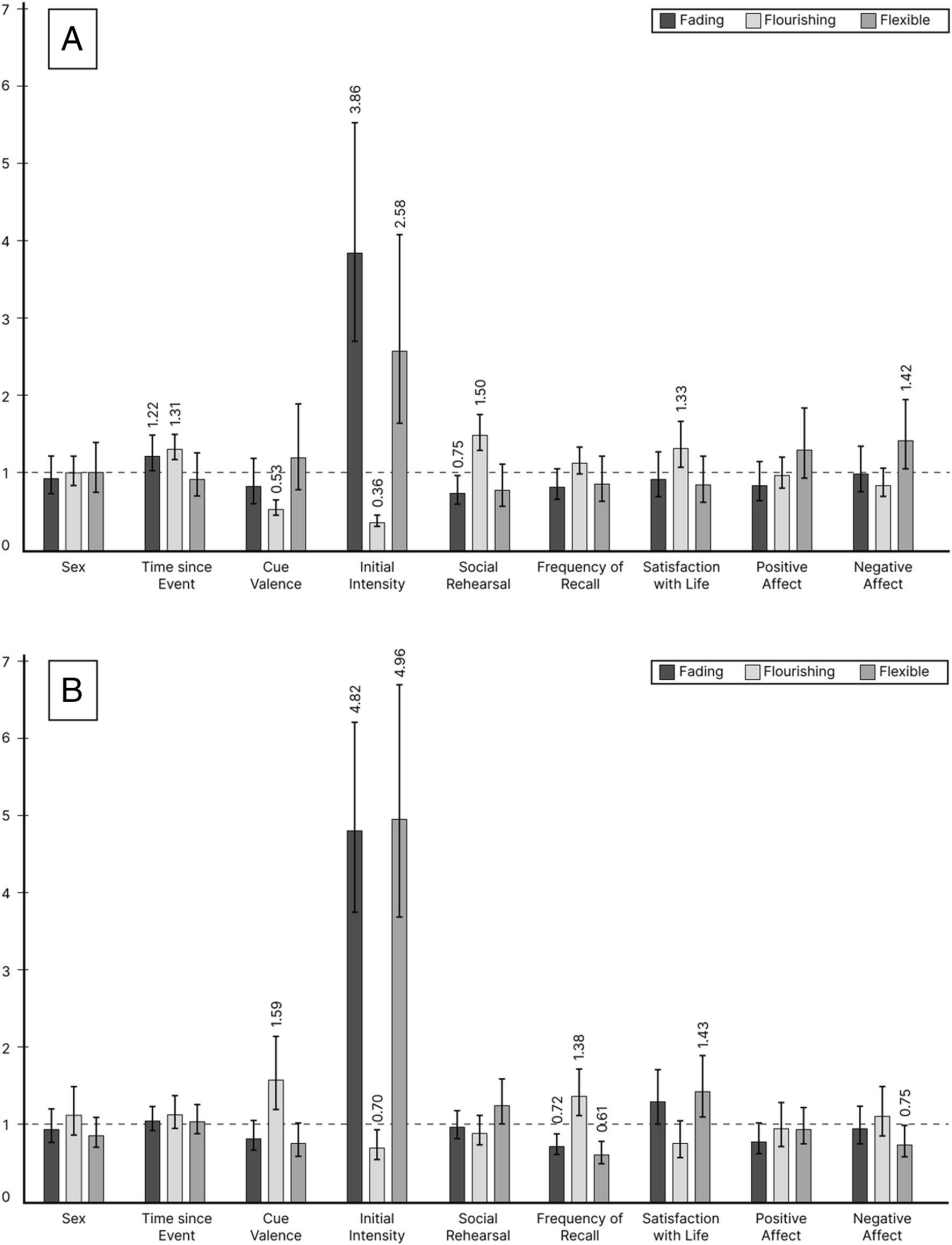
Odds ratio for standardized factor change estimates from the qualitative change model with 95% confidence intervals. **Panel A:** Change in positive emotionality. **Panel B:** Change in negative emotionality

All ORs with 95% confidence intervals are shown in Fig. 1. As can be seen when looking at changes in positive and negative emotionality, and as one would expect, for some predictors the effects go in the same direction, (e.g., higher initial intensity is associated with an increased probability of fading compared to fixed affect in both, positive and negative emotionality), but for other predictors the effects go in opposite directions (e.g., higher individual levels of negative affect are associated with an increased probability of flexible positive but a decreased probability of flexible negative affect compared to staying fixed in affect). In general, the effects of initial intensity were the strongest for both positive and negative change.

### Quantitative change (multilevel multiple regression)

In the second part of the analyses, quantitative change, that is, the amount of change within each category was examined. First, a baseline model (Model 0) was estimated that included only random intercepts. The parameter estimates for the comparisons of the reference category fixed affect with fading, flourishing and flexible affect AMs for both the positive and negative affect models are shown in Table 4.

**Table 4:** Model 0 for quantitative change of positive and negative intensity; Event level: n = 1748; Participant level: N = 117

	Positive Affect Model			Negative Affect Model		
	Fading Positive Affect	Flourishing Positive Affect	Flexible Positive Affect	Fading Negative Affect	Flourishing Negative Affect	Flexible Negative Affect
*Fixed Effects*						
Intercept	−1.449*	1.926*	−1.547*	−1.806*	1.668*	−2.639*
*Random Effects*						
Intercept Variance	0.038	0.274*	3.077*	0.149*	0.242*	0.853*
*Intercept Correlations:*						
Fading Positive Affect	1.000					
Flourishing Positive Affect	−0.589	1.000				
Flexible Positive Affect	−0.035	0.180	1.000			
Fading Negative Affect	0.239	−0.539	−0.312	1.000		
Flourishing Negative Affect	−1.000	0.092	−0.591	−0.276	1.000	
Flexible Negative Affect	−0.152	−0.397	−0.008	0.222	−0.377	1.000
*Model Fit*						
-2LL	7594					
AIC	7640					

^*^ Indicates *p* < .05 (two-tailed); † indicates *p* < .10 (two-tailed)

^a^ Variable is centered between persons

^b^ Variable is centered within persons

Intraclass Correlations Positive Affect Model: .09 (Fading), .40 (Flourishing), .88 (Flexible)

Intraclass Correlations Negative Affect Model: .24 (Fading), .34 (Flourishing), .64 (Flexible)

All intercept estimates were significant. An exemplar interpretation of the intercept estimate *β*_0*FadeP*_ = −1*.*449 for the category of fading positive affect (left half of Table 4) is that the average amount of fading of an AM that showed fading positive affect was a decrease of 1.449 on a scale from *no change* (0) to *very strong change* (7). Participants reliably differed in their mean quantitative change within flourishing and flexible positive affect AMs, as well as within fading, flourishing and flexible negative affect AMs, as indicated by the significant random intercept variances. In the positive affect model, intraclass correlations ranged from .09 (fading positive affect) to .88 (flexible positive affect), indicating that between 9% and 88% of the variance in each model reflected differences between participants. In the negative affect model, intraclass correlations ranged from .24 (fading negative affect) to .64 (flexible negative affect).^8^ The strength of change in some categories was correlated with the strength of change in other categories, as indicated by the intercept correlations in Table 4. For example, participants who reported AMs with relatively strong flourishing of positive affect (increase of intensity) also reported AMs with relatively strong fading of negative affect (decrease of intensity), as indicated by the correlation of −0.539.

In Model 1, control and predictor variables were added, resulting in an increase in model fit (see -2LL and AIC in Table 5). As indicated by the R^2^, the control and predictor variables together accounted for 83% of the variance at the level of AMs, and for 38% of the variance at the level of participants in the positive affect model. Similarly, 85% of the variance at the AM level and 43% of the variance at the participant level was accounted for in the negative affect model. In the following, the results are presented separately for each predictor variable (Table 5).

**Table 5:** Model 1 for quantitative change of positive and negative intensity; Event level: n = 1748; Participant level: N = 117

	Positive Affect Model			Negative Affect Model		
	Fading Positive Affect	Flourishing Positive Affect	Flexible Positive Affect	Fading Negative Affect	Flourishing Negative Affect	Flexible Negative Affect
*Fixed Effects*						
Intercept	−1.247*	1.709*	−0.963*	−1.459*	1.520*	−1.724*
Sex^a^	0.390*	−0.257	−1.337*	0.074	0.011	0.447
Time since the Event^b^	−0.016	0.030*	0.017	−0.025*	0.008	0.008
Cue Valence^b^	−0.123	−0.459*	−0.677*	0.423*	0.449*	0.332*
*Within-Person Vars*						
Initial Intensity^b^	−0.081*	−0.273*	−0.613*	−0.236*	−0.157*	0.565*
Social Rehearsal^b^	0.013	0.170*	0.050	0.006	0.043	0.137*
Frequency of Recall^b^	0.067†	−0.036	−0.170*	0.131*	0.032	0.049
*Between-Person Vars*						
Satisfaction with Life^a^	0.018	−0.064	−0.068	0.020	0.010	−0.209†
Positive Affect^a^	−0.021	0.022	0.312	−0.004	0.170	0.226
Negative Affect^a^	0.030	0.045	−0.289	0.004	0.238*	−0.044
*Random Effects*						
Intercept Variance	0.021	0.295*	1.315*	0.206*	0.275*	0.934*
*Model Fit*						
-2LL	7100					
AIC	7146					
R² within^a^	0.83			0.85		
R² between^b^	0.38			0.43		

^*^ Indicates *p* < .05 (two-tailed); † indicates *p* < .10 (two-tailed)

^a^ Variable is centered between persons

^b^ Variable is centered within persons

^a^ Refers to the proportion of Level 1 (AM) variance explained by the model (see Rights & Sterba, 2019)

^b^ Refers to the proportion of Level 2 (participant) variance explained by the model (see Rights & Sterba, 2019).

#### Event level predictors

The values in Table 5 can be interpreted as follows: the intercept estimate of fading positive affect is negative, indicating that, compared to no change (fixed affect), an AM showing fading has a mean decrease in initial intensity equal to the intercept estimate. The effect of initial intensity is significant and negative, such that (within person) a higher initial intensity is associated with a greater decrease in emotional intensity, that is, an increase in fading.

##### Initial intensity

In the positive affect model, higher initial intensity ratings were associated with stronger fading and weaker flourishing of positive emotional intensity and a greater decrease in positive intensity among AMs demonstrating flexible positive affect. In the negative affect model, higher initial intensity ratings were again associated with stronger fading and weaker flourishing of negative emotional intensity, but a smaller decrease in negative intensity among AMs demonstrating flexible negative affect.

##### Social rehearsal

In the positive affect model, higher levels of social rehearsal were associated with stronger flourishing of positive intensity. In the negative affect model, higher levels of social rehearsal were associated with a smaller decrease in negative intensity among AMs demonstrating flexible negative affect.

##### Frequency of recall

In the positive affect model, higher levels of frequency of recall were associated with a greater decrease in positive intensity among AMs demonstrating flexible positive affect. In the negative affect model, higher levels of recall frequency were associated with weaker fading of negative intensity.

#### Participant level predictors

##### Satisfaction with life

No significant effects of life satisfaction were found.

##### Positive affect

No significant effects of positive affect were found.

##### Negative affect

In the positive affect model, no significant effects of negative affect were found. In the negative affect model, individuals with higher levels of negative affect reported memories with stronger flourishing of negative intensity.

**Fig. 2:**
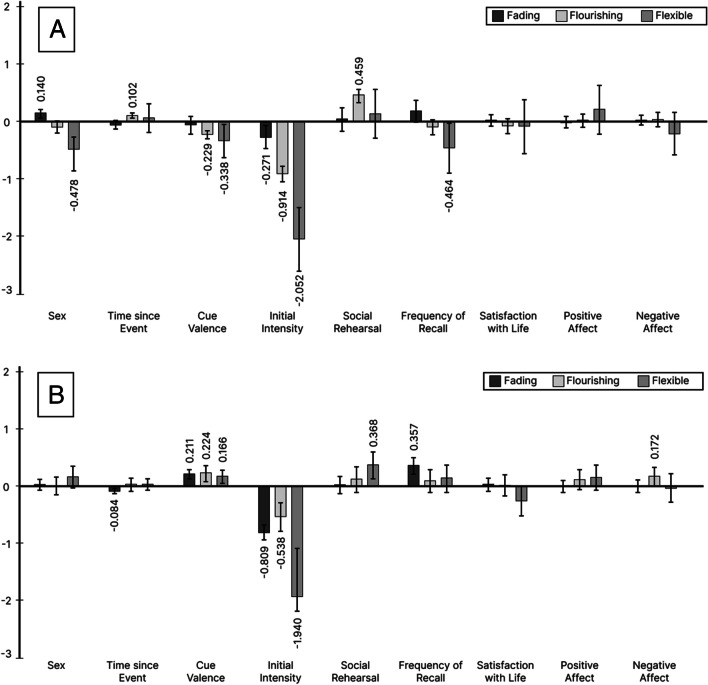
Standardized estimates from the quantitative change model with 95% confidence intervals. **Panel A:** Change in positive emotionality. **Panel B:** Change in negative emotionality

All standardized effects with 95% confidence intervals are shown in Fig. 2. Again, the effects for initial intensity were the strongest for both positive and negative change.

## Discussion

Below we discuss the frequency of the four affect change categories and their amount of emotional change within positive and negative emotionality ratings, followed by the event and participant level predictors.

### Affect change categories across positive and negative emotionality

The present study found frequency biases in all four affect change categories across positive and negative emotionality ratings. Specifically, more AMs showed fading of negative than positive affect, slightly more AMs remained fixed in positive affect than in negative affect, more AMs showed flourishing of positive than negative affect, and finally, more AMs changed from negative to positive valence than vice versa. The present findings are consistent with previous research investigating the frequency of the four affect change categories (Gibbons & Rollins, 2016; Hoehne, 2023; Ritchie et al., 2009).

The reader may note that most studies that have examined the FAB to date have not compared the frequency of fading across positive and negative emotionality, but rather the amount of fading in positive and negative emotional intensity (e.g., Muir et al., 2015; Ritchie et al., 2006; Walker et al., 2020). In order to extend the current understanding of the biases in the affect change categories other than fading affect, the present study additionally examined the amount of emotional change within each category across positive and negative emotionality. Complementing the frequency biases, the present findings revealed biases in the strength of change. Specifically, there was a greater decrease in emotional intensity among AMs that showed fading negative affect compared to fading positive affect. Similarly, there was a greater increase in emotional intensity for AMs that showed flourishing positive affect than for those that showed flourishing negative affect. Finally, when an AM changed from negative to positive valence, the amount of change was greater than when it changed from positive to negative valence. Thus, the present study adds to research on the FAB by demonstrating that not only fading affect, but also fixed, flourishing, and flexible affect show biases based on (a) the frequency and (b) the amount of change of each category within positive and negative emotionality. The first hypothesis of the present study, that frequency biases as well as biased in the amount of change would be evident for all four affect change categories, was therefore fully supported by the present data.

The emergence of biases in flourishing, fixed, and flexible affect, apart from the FAB shows that in order to improve our understanding of emotional change and also emotion regulation processes in autobiographical memory, a more detailed examination of the types of emotional change is required, rather than grouping them into one phenomenon, the FAB. In particular, flexible affect, i.e., a change in valence, is an informative type of emotional change in AMs, as it shows that primarily negative events are not fully taken into account when it is demonstrated that they lose their emotional intensity to a greater extent and at a faster rate than primarily positive events. In the present study, about 13% of all AMs were rated as having changed from negative to positive, which is obviously more than just a decrease in negative intensity. In comparison, only about twice as many AMs were rated as having faded in negative intensity. The processes that shape our autobiographical memory are not fully accounted for when we reduce them to a narrative of fading, and future research may take this into account.

### Predictors of the likelihood and strength of emotional change in AMs

#### Initial intensity

The second hypothesis of the present study was also fully supported by the present data, as for both, the positive and the negative affect models, AMs with low compared to high initial intensity ratings had a reduced likelihood of exhibiting fading and flexible affect, and an increased likelihood of exhibiting flourishing affect compared to remaining fixed in affect. These findings are consistent with those of Hoehne (2023), who used the same operationalization of the different affect change categories but a different type of memory elicitation. Most relevantly, flexible affect AMs again showed higher initial intensity ratings than fixed affect AMs in the present study. It thus seems that flexible affect AMs should be considered as more than just a change on a bipolar scale from positive to negative or vice versa, which becomes more likely the closer the initial event intensity is to neutral like it was found in Gibbons and Rollins (2016). Because it involves a change in valence, flexible affect may be one of the most informative types for understanding emotional change in AMs, and therefore its operationalization should be carefully considered in future research. The reader may note that an ongoing debate concerns the assumption that positive and negative affect represent a bipolar continuum versus that they can be perceived as two dimensions (e.g., Larsen & Green, 2013). Diener and Emmons (1985) argued that, especially when positive and negative affect are examined over time, they represent distinctly different dimensions of human emotional experience, indicating that both can be associated with the same event.

When examining the strength of change within each category, in both models, high initial intensity ratings were associated with stronger fading and weaker flourishing, which can be explained by ceiling and floor effects. However, high initial intensity ratings were associated with a stronger decrease in positive emotional intensity within the category of flexible positive affect, but a weaker decrease in negative emotional intensity within the category of flexible negative affect. One possible interpretation would be that reappraising a highly negative event in a positive light does not necessarily mean that one forgets or represses all the negative aspects of the event, but rather that one finds positive meaning without forgetting the negative. In some regards, this finding is consistent with studies who emphasizes the importance of the coexistence of both the positive and the negative in order to function and flourish (e.g., Ivtzan et al., 2015; Lomas & Ivtzan, 2016), instead of focusing only on the positive and excluding anything that could be considered negative.

#### Social rehearsal

Higher levels of social rehearsal were associated with a decreased likelihood of fading positive affect and an increased likelihood of flourishing positive affect compared to remaining fixed in positive affect, whereas no effects were found in the negative affect model. Most studies that have investigated social rehearsal in the context of emotional change in AMs have (a) focused on its moderating role only on the FAB and (b) used only one scale to measure emotionality (e.g., Skowronski et al., 2004). As described in the introduction, Skowronski et al. (2004) could not distinguish whether their findings were due to a decrease in negativity, an increase in positivity, or both. The present study used separate scales to investigate the relationship of social rehearsal to emotional change in AMs, and replicates what was found in Hoehne (2023): Consistent with Muir et al.’s (2019) finding that positive facilitation (rather than negative inhibition) is a relevant aspect of listeners’ responses to increase FAB following social disclosure, the benefits of social rehearsal appear to work through an increase of positive emotionality, not a decrease in negative emotionality.

Specifically, the following processes may take place when communicating an AM to others. Narrating the AM first gives some structure to the memory, as it requires a certain amount of reflection (Skowronski et al., 2014). During disclosure, the person sharing feels validated when a listener provides backchanneling and demonstrates understanding (Muir et al., 2019). Finally, after disclosure, the listener may facilitate a positive interpretation of the event by providing positive feedback (Muir et al., 2019; Skowronski et al., 2014). All of these processes might help to increase the positive emotionality of one’s own emotional evaluation of the event.

In addition, the present findings can also be interpreted in connection to theories of emotional memory. For instance, it is suggested that a negative emotional evaluation of an event is associated with a narrowed focus of attention. That is, when negative events are remembered, bottom-up processes are likely and specific details of the situation are recalled and analyzed. However, recalling positive events is associated with a broadened focus of attention, characterized by top-down processes and a more global, general view of the world, leading to a more reconstructive memory (Smorti & Fioretti, 2016). One effect of social rehearsal is that through sharing and feedback, some sort of structure is given to the memory (Skowronski et al., 2014), as sharing an event requires an explanation, for which the memory needs to be elaborated in a coherent story (Smorti & Fioretti, 2016). This, in turn, may help the memory to be integrated in the bigger picture. Consequently, according to this theory, sharing an event may lead to a broadened focus from which the event is recalled, which may increase positivity. Contrary to previous theories, the present findings did not support that a broadened focus by sharing an event counteracts the perception of its negative aspects (Smorti & Fioretti, 2016), as social rehearsal only affected changes in positive, but not negative emotionality. Rather, it is suggested that the integration into the bigger picture helps to understand the event and therefore increases positivity, but without losing memory for the negative details of the situation.

Social rehearsal also showed significant effects on the amount of change within the categories. Supporting and extending the findings from the probability model, higher levels of social rehearsal were associated with a greater increase in positive emotional intensity among flourishing positive affect AMs. Furthermore, sharing AMs was associated with a smaller decrease in negative emotional intensity among AMs demonstrating flexible negative affect. The reader may note that there was a tendency for a higher frequency of social rehearsal to be associated with a higher likelihood of an AM exhibiting flexible negative compared to fixed negative affect. Taken together, these findings suggest the following mechanism: Communicating a negative AM to others helps to reframe the event in a positive light, integrate it into the bigger picture and to find the positive aspects of the situation without forgetting its negative aspects, which could be an indicator of healthy coping, and serves as further evidence that social rehearsal promotes positivity but does not reduce negativity. Therefore, also the third hypothesis of the present article was supported by the present data. Importantly, apart from Hoehne (2023), this is the first study to examine the influence of social rehearsal on AMs’ emotional change by using separate scales for positive and negative emotionality. The results are revealing in two respects. First, they clarify the picture of how social rehearsal affects emotional change in AMs, and second, they support the view that positive and negative emotionality in AMs are bidirectional and function differently.

#### Frequency of recall

Frequency of recall showed significant effects on the likelihood of the affect change categories, but only in the negative affect model. Specifically, a high frequency of recall of an AM was associated with a reduced likelihood of that AM showing fading and flexible negative affect, and an increased likelihood of it showing flourishing negative affect compared to remaining fixed. Therefore, the fourth hypothesis, that higher recall frequency is associated with lower fading of negative intensity, was also supported by the present data. The present findings fit the most commonly found pattern that a high recall frequency is associated with a reduced FAB (Muir et al., 2022; Ritchie et al., 2006, 2015; Walker et al., 2009). In accordance, the present study found that high levels of frequency of recall not only reduced the likelihood of an AM exhibiting fading negative affect, but also reduced the amount of fading among those exhibiting fading negative affect. In addition, thinking about an event was associated with a greater decrease in positive emotional intensity among AMs exhibiting flexible positive affect. In summary, the present results replicate and extend what has previously been found regarding frequency of recall, however, instead of examining only fading affect, the present study shows that frequency of recall also affects other types of emotional change in AMs. The present findings support the contention that thinking (i.e., frequently recalling) about events is a maladaptive way of coping with life events, as it has no benefits for positive AMs but actually increases negativity for negative AMs.

#### Subjective wellbeing

The FAB has already been linked to several maladaptive individual traits that have been found to reduce the FAB, such as depression (Walker et al., 2003), anxiety (Walker et al., 2014), and narcissism (Ritchie et al., 2015), but it has also been linked to grit, a positive individual trait that has been found to increase the FAB (Walker et al., 2020). In the present study, life satisfaction, the cognitive-evaluative component of SWB, was associated with an increased likelihood of an individual reporting flourishing positive affect AMs and also with an increased likelihood of reporting AMs that changed from negative to positive instead of reporting fixed negative affect AMs. This is in line with the proposition that adaptive personality traits lead to more positive changes in an individual’s AMs. Interestingly, positive affect, the affective, adaptive part of SWB, showed no effects when the other predictors were included, suggesting that the cognitive evaluation of our life situation is more relevant for our evaluation of the emotional change in AMs. This finding may also be explained by the fact that in the present study participants were asked to rate the emotionality of events at occurrence retrospectively, thus provoking a more cognitive evaluation of the event.

Negative affect, however, which is the maladaptive, affective component of SWB, showed significant effects that took the form of a reduction in positivity and an increase in negativity. Specifically, high levels of negative affect were associated with an increased likelihood of the individual reporting AMs that changed from positive to negative, but a decreased likelihood of reporting AMs that changed vice versa. Furthermore, negative affect was also associated with a greater increase in negative emotional intensity among AMs exhibiting flourishing negative affect. Therefore, the fifth hypothesis, that higher levels of individual wellbeing would be associated with an increase in positivity and a decrease in negativity, was also supported. Taken together, these findings support and extend previous research on personality traits and the FAB. In addition, because the present study did not solely focus on fading affect, a more detailed examination of how individual characteristics are related to each of the affect change categories was possible.

### Limitations and future directions

The present study used a retrospective method, which only allowed for the investigation of *perceived* emotional change as opposed to actual emotional change. Ritchie et al. (2009) were able to show that the FAB cannot be explained by retrospective bias when retrospective emotionality ratings are compared with diary ratings of the same event. However, Ritchie et al. (2009) only used a time difference of about two weeks to compare diary and retrospective ratings. In the present study, however, the reported events were much older. It is therefore important to consider several possible mechanisms of retrospective recall when interpreting the results. One example of this is a person’s current mood. Being in a bad mood could lead to a temporally increased negative focus on past events, while being in a good mood could lead to a temporally increased positive focus on past events. Moreover, retrospectively rating the emotionality of an event might require more cognitive elaboration of the event than rating one’s current emotions at the moment of the event. It is therefore possible that the present finding that life satisfaction (cognitive evaluation), but not positive affect (emotional evaluation), was associated with changes in AMs’ emotionality was an artefact of the retrospective methodology. However, because AMs are shaped by current feelings, goals, or ideas about the self (Conway & Pleydell-Pearce, 2000), they are subjective, and it could be argued that perceived emotional change in AMs is more relevant than actual emotional change because it reflects what people remember, which never is objective. An interesting research question for future research might be to investigate whether the perceived emotional change in an AM remains stable over time. For example, participants could be asked to rate their perceived emotional change in their AMs again after a year or two to examine their stability.

Data collection in the present study took place online, which has the advantage that participants may be more open in reporting personal memories than when reporting to a researcher in person. However, online data collection also has the disadvantage of reducing control over how the study was conducted. Also, the type of memory sampling may have influenced the present findings. Different types of memory sampling can lead to different sets of memories in terms of memory characteristics, and the present results show, that the type of change in an AM is associated with such characteristics. For instance, asking only about important memories, might lead to a sample of highly intense AMs, which in turn might lead to different frequencies of the different affect change categories. The present study replicated the findings from Hoehne (2023), who used a different type of memory sampling. Of course, however, this, is not a systematic comparison.

For data analysis, the present study chose multilevel modeling to account for the data dependency which arises when participants report more than one AM. The present study shows a considerable amount of data dependency, as indicated by the intraclass correlations. Ignoring this data dependency, as most previous studies of the FAB have done, can easily lead to biased parameter estimates. Furthermore, the use of multilevel models allowed for the simultaneous examination of predictors at the level of AMs (e.g., social rehearsal) and at the level of the participant (wellbeing), which is another advantage of the present analysis technique. Statistically, the present model could have been extended in several ways. For instance, it would have been possible to include random slope effects or cross-level interactions. To illustrate, we could have examined whether the strength of the effect of social rehearsal on AMs’ emotional change differed between participants, and whether this could have been explained by a specific characteristic of the individual. However, there is currently no theoretical basis for such mechanisms that would justify the inevitably increases in complexity that would result, so such extensions were not included.

The present sample consisted mainly of female participants, and previous studies have sometimes found that female participants reported more emotional AMs than men (e.g., Grysman et al., 2017; Grysman & Hudson, 2013; Seidlitz & Diener, 1998). Moreover, previous studies have also found that women perform better at emotion recognition than men, but worse at regulating unpleasant emotions (e.g., Yuan et al., 2010). It is not clear how these different processes might differentially influence AMs’ affective change in men and women. In the present results, however, gender at most influenced the strength of change with respect to fading and flexible positive affect, but not the likelihood of any of the affect change categories. On average, male participants reported a weaker fading of positive affect, but a stronger decrease in positive intensity in AMs showing flexible positive affect. This finding speaks for the validity of the results, despite the gender imbalance in the present sample. Moreover, the present study replicated the main findings of Hoehne (2023), who used a more gender-balanced sample, which also makes it seem unlikely that the present results are biased by the gender imbalance of the participants. Notwithstanding, future research could explore more deeply the role of gender in AMs’ emotional change and its predictors.

Finally, future research could explore different operationalizations as well as additional predictors of the affect change categories. The present study found that when participants were asked to actively choose whether an AM showed flexible affect, the results differed from studies that used difference scores (Gibbons & Rollins, 2016). Future studies could operationalize each type of affect change, as was done for flexible affect in the present study, and investigate whether the present findings still hold. This way of operationalizing the different affect change categories would also allow initial intensity to be examined without the influence of ceiling- and floor effects, which may have strongly influenced the present findings on initial intensity. Another possible operationalization of AMs’ emotional change could be the use of multiple emotion scales, that go beyond positive and negative emotionality (i.e., happiness, anxiety, or satisfaction) to more fully explore emotional change. Moreover, all of the predictors examined in FAB research could be further explored by examining their association with all types of emotional change in AMs. Specifically, it might be informative whether and which types of emotion regulation are associated with specific changes in AMs, as emotion regulation, defined as all “processes by which individuals influence which emotions they have, when they have them, and how they experience and express these emotions” (Gross, 2015, p.275), is strongly related to a change in emotions.

## Conclusion

The present study provided further evidence for the existence of frequency biases across positive and negative emotionality ratings for all types of emotional change in AMs, and even extended this finding by providing evidence that the biases also apply to the amount of change within each affect change category. Furthermore, the present study was able to replicate Hoehne’s (2023) findings regarding initial intensity and social rehearsal and also extended them, by including recall frequency and SWB as additional predictors and by examining the influence of all predictors on the amount of emotional change within each category. Finally, the present study provides a valuable analytical framework for a relatively unexplored research topic within the field of autobiographical memory research. Emotional change in AMs warrants further investigation, as how we see ourselves and the world is largely defined by our autobiographical memory.

## Data Availability

The dataset generated during and/or analyzed during the current study is available in the OSF repository, https://osf.io/puye2/. The present study was not preregistered.

## Footnotes

1 To determine the necessary sample size, typically an a priori power analysis is conducted. However, both the analysis model and the data structure defy any straightforward power analysis. Therefore, in estimating the necessary sample size, our aim was to have enough observations (ca. 50 AMs) even in the smallest affect change category (flexible positive affect). Based on previous studies (e.g., Gibbons & Rollins, 2016; Hoehne, 2023; Ritchie et al., 2009), we calculated with 2–3% AMs showing flexible positive affect, such that an event level sample size of approximately 1,700 AMs resulted. To have a sufficient number of AMs per participant to calculate between-person effects, while at the same time trying to limit the load on participants in the present study, we decided to ask each participant to report up to 16 AMs. This resulted in a sample size of approximately 106 participants.

2 The cues were previously tested for emotional intensity and likelihood of having been experienced in another unpublished study. Some cues were replaced from the original set of cues due to their potentially low self-relevance. Emotion-regulation processes may be particularly evident for self-relevant information.

3 Indeed, the four affect change categories can be ordered according to direction and strength of change in AMs’ emotional intensity. An alternative approach would, thus, be to use an ordered logit model (e.g., McCullagh, 1980), which would treat the categories either in a descending or ascending order (Zimprich & Wolf, 2018). However, treating the four affect change categories as ordered would also imply that the different AM characteristics are ordered across the categories. To avoid these restrictions through ordering, we chose a multinomial approach, which is able to treat the different categories as nominal (e.g., Long, 1997).

4 The reader may note that while the qualitative part required the estimation of two separate models for change in positive and change in negative emotionality, in the quantitative part both could be estimated in one model.

5 Transformed back to the probability one gets

$$\begin{array}{lc}{p}_{FadeP}=\frac{{\mathrm{exp}}\left({\beta }_{0FadeP}\right)}{1+\mathit{exp}\left({\beta }_{0FadeP}\right)+{\mathrm{exp}}\left({\beta }_{0FlourishP}\right)+{\mathrm{exp}}\left({\beta }_{0FlexibleP}\right)}\\\;\;\;\;\;\;\;\;\;\;\;= \frac{exp(-1.566)}{1+\mathit{exp}\left(-1.566\right)+\mathit{exp}\left(-0.443\right) +\mathit{exp}\left(-2.761\right)}= .109.\end{array}$$

6 In generalized linear mixed models, marginal and conditional models are distinguished. The observed (marginal) distribution of the outcome variable is different from the distribution of the outcome variable conditional on the random effects (Stroup, 2012). With increasing random variance, the difference increases between these two distributions.

7 A useful calculation to interpret and compare the estimates is transforming them into an odds ratio (OR) for a standardized factor change (Long, 1997). For the effect of initial intensity (centered within-person) on fading positive affect one gets

$$\begin{array}{l}OR=exp\left(\beta _{\mathrm{InitialIntensityFadeP}}\times SD{_{\mathrm{InitialIntensity^{b}P}}}\right)\\\;\;\;\;\;\;\; ={\mathrm{exp}}\left(0.572 \times 2.36\right)=3.86\end{array}$$

indicating that for a standard deviation change in initial intensity, the odds for this AM to show fading compared to fixed positive affect increase by a factor of 3.86.

8 The reader may note that the intraclass correlations for flexible affect are quite high in both the positive and the negative affect models, indicating that the variance in flexible affect is mainly due to differences between participants. The reason for this is that many participants reported no or very few flexible affect AMs, while a few participants reported a high number of flexible affect AMs.

## Code availability

The SAS syntax associated with the present work is available in the OSF repository, https://osf.io/puye2/.
